# On variational solutions for whole brain serial-section histology using a Sobolev prior in the computational anatomy random orbit model

**DOI:** 10.1371/journal.pcbi.1006610

**Published:** 2018-12-26

**Authors:** Brian C. Lee, Daniel J. Tward, Partha P. Mitra, Michael I. Miller

**Affiliations:** 1 Center for Imaging Science, Department of Biomedical Engineering, Johns Hopkins University, Baltimore, MD, USA; 2 Cold Spring Harbor Laboratory, Cold Spring Harbor, NY, USA; University of Pennsylvania, UNITED STATES

## Abstract

This paper presents a variational framework for dense diffeomorphic atlas-mapping onto high-throughput histology stacks at the 20 μm meso-scale. The observed sections are modelled as Gaussian random fields conditioned on a sequence of unknown section by section rigid motions and unknown diffeomorphic transformation of a three-dimensional atlas. To regularize over the high-dimensionality of our parameter space (which is a product space of the rigid motion dimensions and the diffeomorphism dimensions), the histology stacks are modelled as arising from a first order Sobolev space smoothness prior. We show that the joint maximum a-posteriori, penalized-likelihood estimator of our high dimensional parameter space emerges as a joint optimization interleaving rigid motion estimation for histology restacking and large deformation diffeomorphic metric mapping to atlas coordinates. We show that joint optimization in this parameter space solves the classical curvature non-identifiability of the histology stacking problem. The algorithms are demonstrated on a collection of whole-brain histological image stacks from the Mouse Brain Architecture Project.

## Author summary

New developments in neural tracing techniques have motivated the widespread use of histology as a modality for exploring the circuitry of the brain. Automated mapping of pre-labeled atlases onto modern large datasets of histological imagery is a critical step for elucidating the brain’s neural circuitry and shape. This task is challenging as histological sections are imaged independently and the reconstruction of the unsectioned volume is nontrivial. Typically, neuroanatomists use reference volumes of the same subject (e.g. MRI) to guide reconstruction. However, obtaining reference imagery is often non-standard, as in high-throughput animal models like mouse histology. Others have proposed using anatomical atlases as guides, but have not accounted for the intrinsic nonlinear shape difference from atlas to subject. Our method addresses these limitations by jointly optimizing reconstruction informed by an atlas simultaneously with the nonlinear change of coordinates that encapsulates anatomical variation. This accounts for intrinsic shape differences and enables rigorous, direct comparisons of atlas and subject coordinates. Using simulations, we demonstrate that our method recovers the reconstruction parameters more accurately than atlas-free models and innately produces accurate segmentations from simultaneous atlas mapping. We also demonstrate our method on the Mouse Brain Architecture dataset, successfully mapping and reconstructing over 1000 brains.

## Introduction

### Mapping brain circuitry

Recent advances in brain imaging [1, 2], methods to label neurons [3], and computational methods have brought about a new era of neuroanatomical research, with a focus on comprehensively mapping brain circuits [4]. Mapping whole-brain circuitry is important for three distinct reasons: scientific understanding of how the brain works, mechanistic understanding of neurological and neuropsychiatric disorders, and as a comparison point for artificial neural networks used in machine learning [5, 6].

Circuit mapping is technique limited, and falls into three broad scales corresponding to distinct imaging modalities—indirect mapping at a macroscopic scale corresponding to MRI-based methods [7], and direct mapping at light (LM) and electron microscopic (EM) scales. For MRI and LM data, atlas mapping is an important step in the analysis. Several approaches exist for gathering LM data at the whole brain level [8–10]. For some of these approaches (two-photon serial block-face imaging, knife edge scanning microscopy and light sheet microscopy for cleared brains) two-dimensional (2D) optical sections are acquired in three-dimensional (3D) registry with each other, so that the only computational step required is 3D volumetric registration of the individual brain data set to a canonical atlas. However, for classical neurohistological approaches using tissue sectioning followed by histochemical processing, the 2D sections are gathered independently and each section can undergo an arbitrary rotation and translation compared to the block face. This may be considered a disadvantage of the classical neuroanatomical workflow, however the physical sectioning method followed by conventional histochemical analysis has certain important advantages. This allows for the full spectrum of histochemical stains, acquisition of physical sections for downstream molecular analyses, and processing for larger brains (upto and including whole human brains). Therefore it is necessary to perform an intermediate 2D to 3D registration step, where the individually acquired 2D sections are mutually co-registered into a 3D volume.

This paper develops a joint stack reconstruction and atlas mapping procedure that simultaneously restacks the 2D histology sections, applying a sequence of rigid motions to the sections, and estimates the diffeomorphic correspondence between the registered histology stack and the 3D atlas. We apply these algorithms to data sets from the Mouse Brain Architecture Project (MBAP), for which the experimental workflow generating the data utilizes a tape transfer technique [11], allowing for the sections to maintain geometrical rigidity within section and also allowing for physically disjoint components to maintain their spatial relations. The tape method ensures that the number of missing sections is minimal, with serial sections cut at a thickness of 20 μm and alternate sections subjected to Nissl staining alongside staining with histochemical or fluorescent label. These Nissl stained sections form the basis of alignment to a Nissl whole-brain reference atlas.

### Computational anatomy methods for brain histology

The histological reconstruction problem has been explored by several groups previously. Malandain first described the ill-posedness of reconstructing 3D sections and object curvature without prior knowledge of the shape of the object [12]. Rigid transformations for stack reconstruction have been estimated via block-matching of histological sections in [13], with point information based on landmarks introduced to guide volume reconstruction [14]. Dense external reference information such as MRI has been applied to guide reconstruction via registration of corresponding block-face photographs and for histology to MRI mapping [15, 16].

The principal contribution of this work is to rigorously solve the problem when an external resource of identical geometry (such as an MRI of the same mouse) is not available, while accommodating for the innate anatomical variation from atlas to subject. The lack of a same-subject reference volume is often the standard in mouse brain histology and other large scale histology studies. This places us into the computational anatomy (CA) orbit problem for which constraints are inherited from an atlas that is diffeomorphic but not geometrically identical. With the availability of dense brain atlases at many resolution scales [17–20], methods to map atlas labels onto target coordinate systems are being ubiquitously deployed across neuroscience applications. Since Christensen’s early work [21], diffeomorphic transformation has become the de-facto standard as diffeomorphisms generate one-to-one and onto correspondences between coordinate systems. Herein we focus on the diffeomorphometry orbit model [22, 23] of computational anatomy [24], where the space of dense volume imagery is modelled as a Riemannian orbit of an atlas under the diffeomorphism group. We use the large deformation diffeomorphic metric mapping (LDDMM) algorithm first derived for dense imagery by Beg [25] to retrieve the unknown high-dimensional reparameterization of the template coordinates.

Of course, for the histological stacking problem solved here, the interesting twist is the augmentation of the random orbit model with 3 rigid motion dimensions for each target section. At 20 μm, this implies as many as 500 sections augmenting the high-dimensionality of the diffeomorphism space to include as many as 1500 extra dimensions for planar rigid motions for restacking. Here lies the crux of the challenge. To accommodate the high-dimensionality of the unknown rigid motions, the space of stacked targets is modelled to have finite-squared energy Sobolev norm, which enters the problem as a prior distribution restricting the roughness of the allowed restacked volumes. The variational method jointly optimizes over the high-dimensional diffeomorphism associated to the atlas reparameterization and the high-dimensional concatenation of rigid motions associated to the target.

## Materials and methods

### The log-likelihood model of the histology sectioning problem

**Fig 1:**
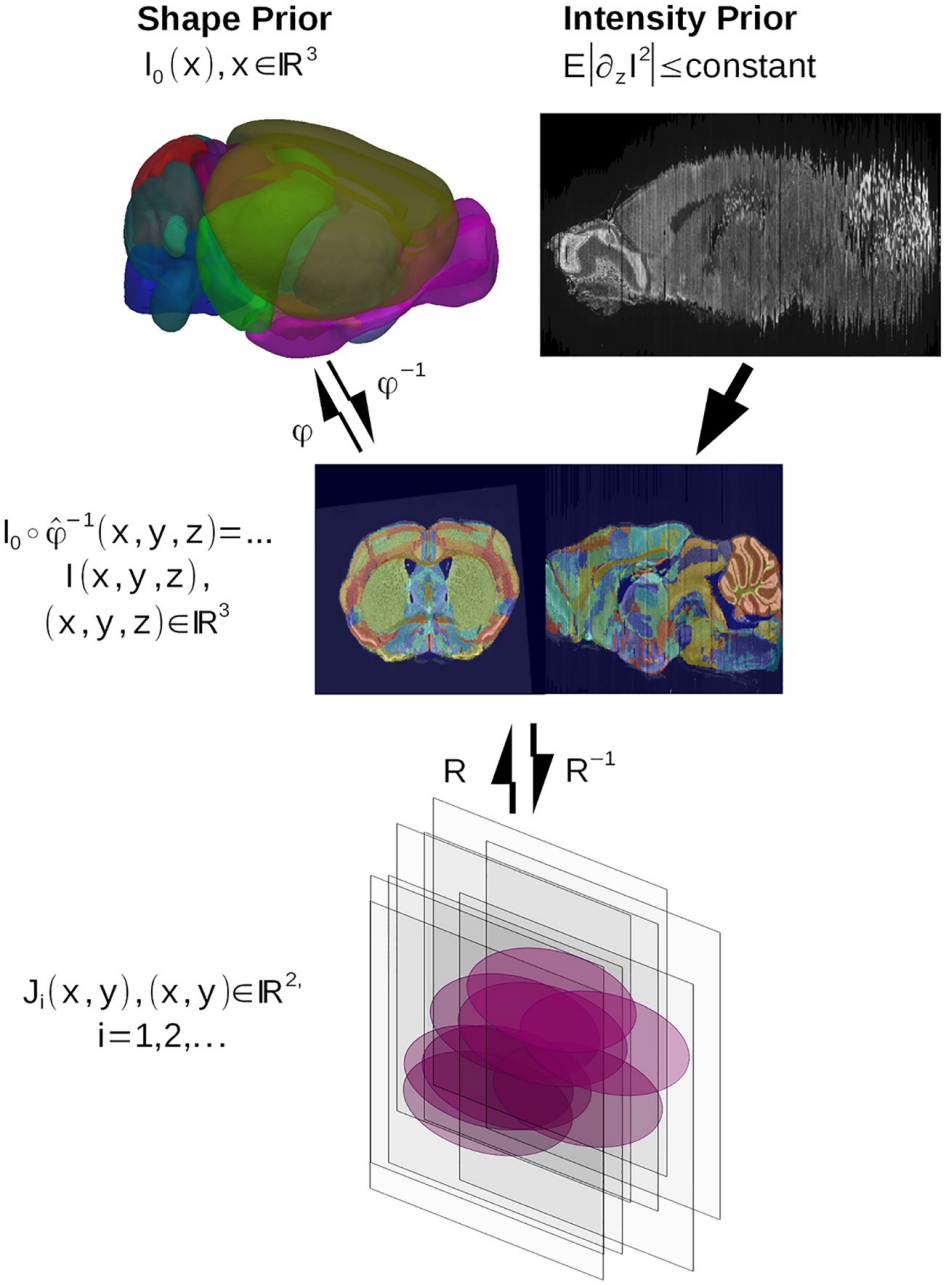
The histological sectioning model. The template *I*_0_, the mouse brain in the orbit I∈I and observed histological sections *J*_*i*_, *i* = 1, …, *n* are illustrated. The Sobolev image intensity prior and the shape prior are depicted in the top row. The model shows the template and mouse brain as elements of the same orbit $I_{0},I\in I$, such that there exists diffeomorphism *I* = *I*_0_ ∘ *φ*^−1^, *φ* ∈ *Diff*.

Fig 1 shows the components of the model for the histology stacking problem. We define the mouse brain to be sectioned as a dense three-dimensional (3D) object $I\left(x,y,z\right),\left(x,y,z\right)\in R^{3}$, modelled to be a smooth deformation of a known, given template *I*_0_ so that *I* = *I*_0_ ∘ *φ*^−1^ for some invertible diffeomorphic transformation *φ*. The Allen Institute’s mouse brain atlas [26] (CCF 2017) is taken as the template. Distinct from volumetric imaging such as MRI which delivers a dense 3D metric of the brain, the histology procedure (bottom row, Fig 1) consisting of sectioning, staining, and imaging generates a jitter process which randomly translates and rotates the stack sections. Denote the rigid motions acting on the 2D sectioning planes $R_{i}:R^{2}\to R^{2}$,

$$\begin{array}{c} R_{i}\left(x,y\right)=\left(\cos\theta_{i}x+\sin\theta_{i}y+t_{i}^{x},-\sin\theta_{i}x+\cos\theta_{i}y+t_{i}^{y}\right)\;,\;\left(x,y\right)\in R^{2}\;, \end{array}$$ (1)

with *θ*_*i*_ the rotation angle and $\left(t_{i}^{x},t_{i}^{y}\right)\in R^{2}$ the translation vector in section *i*. The histology stack $J_{i}\left(x,y\right),\left(x,y\right)\in R^{2},i=1,\ldots ,n$, is a sequence of 2D image sections with jitter under smooth deformation of the atlas in noise:

$$\begin{array}{c} J_{i}\circ R_{i}\left(x,y\right)=I_{0}\circ \varphi^{-1}\left(x,y,z_{i}\right)+noise\left(x,y\right),\;\;\left(x,y\right)\in R^{2}\;. \end{array}$$ (2)

Modeling the photographic noise as Gaussian and conditioning on the sequences of jitters *R*_*i*_, *i* = 1, …, *n* and atlas deformation *I* = *I*_0_ ∘ *φ*^−1^, *φ* ∈ *Diff*, the photographic sections *J*_*i*_ are a sequence of conditionally Gaussian random fields with log-likelihood

$$\begin{array}{ccc} \ell (v,R;J) & = & \sum_{i}(-\alpha_{i}\int_{R^{2}}{\left|J_{i}\circ R_{i}\left(x,y\right)-I_{0}\circ \varphi^{v,-1}\left(x,y,z_{i}\right)\right|}^{2}dxdy). \end{array}$$ (3)

Here *α*_*i*_ is a weighting factor dependent on the noise of each section such that damaged sections can be weighted; *v* denotes the vector field which indexes the deformation as a diffeomorphic flow (see below).

### The priors: Diffeomorphisms and Sobolev smoothness of images

The parameterization of the histology pipeline augments the standard random orbit model of computational anatomy with the rigid-motion dimensions of the random jitter sectioning process. The unknowns to be estimated become $\left(R_{1},\ldots ,R_{n},\varphi \right)\in R^{3n}\times Diff$ for *n*−sections. At 20 μm then *n* = 500 implying the nuisance rigid motions are of high dimension *O*(1500). The solution space must be constrained. We use priors on the deformations and on the rigid motion stacking of the images.

#### The diffeomorphism prior

The histological stacking constrains the brains as smooth transformations of the template, where the diffeomorphisms are generated as diffeomorphic flows *φ*_*t*_ ∈ *Diff* [24], solving the ordinary differential equation

$$\begin{array}{c} {\dot{\varphi }}_{t}=v_{t}\circ \varphi_{t},t\in \left[0,1\right],\;\varphi_{0}=identity\;, \end{array}$$ (4)

with *v*_*t*_ the Eulerian velocity taking values in $R^{3}$, *identity* the identity mapping. The top row of Fig 1a shows that each *φ* has an inverse and that the random orbit model assumes any individual brain I∈I can be generated from the exemplar under the action of the diffeomorphism, so that for some *φ* ∈ *Diff*, *I* = *I*_0_ ∘ *φ*^−1^.

To score the distances between mouse brain coordinate systems and reject outlier solutions we use geodesic flows minimizing metric length [27]. Large deviations as measured by the diffeomorphometry metric [22] from template atlas to target mouse brain are thus removed from the solution space. The vector fields are modeled to be in a reproducing kernel Hilbert space (RKHS) (*V*, ‖⋅‖_*V*_), supporting one continuous spatial derivative, and having geodesic length between coordinate systems determined by the norm-square ${\left\|v\right\|}_{V}^{2}$ of the RKHS:

$$\begin{array}{c} {\left\|v\right\|}_{V}^{2}=\sum_{i=1}^{3}\int_{R^{3}}{\left({\left(-\nabla^{2}+1\right)}^{2}v_{i}\left(x,y,z\right)\right)}^{2}dxdydz<\infty \;. \end{array}$$ (5)

This square-metric is used as a quadratic potential for the smoothness prior between images $I,I^{\prime }\in I$ [28, 29] minimizes the action

$$\begin{array}{c} \rho^{2}\left(I,I^{\prime }\right)=\min_{\varphi :\varphi_{0}=id\;,\varphi_{1}\cdot I=I^{\prime }}\int_{0}^{1}{\left\|v_{t}\right\|}_{V}^{2}dt\;. \end{array}$$ (6)

See S2 Text for the explicit equations for geodesics satisfying the Euler-Lagrange equations [27, 30] and S1 Text for the matrix Green’s kernel.

We use the notation *φ*^*v*^ to emphasize the dependence of the diffeomorphism and the geodesic metric on the vector field *v*. Strictly speaking, the group generated by integrating (4) with finite norm ‖⋅‖_*V*_ is both dependent on the norm of *V* as well as a subgroup of all diffeomorphisms; we shall suppress that technical detail in the notation.

#### The prior distribution on image smoothness

To score the maximum a-posteriori (MAP) reconstruction of the rigid motions acting on the stack, we exploit a smoothness prior on the reconstructed histology stack which enforces the fact that anatomical structures are smooth and continuous. We model the images as arising from a smooth “Sobolev” or RKHS *I* ∈ *H*^*k*^ supporting derivatives $\partial_{h}f=\frac{\partial^{h_{1}+h_{2}+h_{3}}}{\partial x^{h_{1}}\partial y^{h_{2}}\partial z^{h_{3}}}f$ that are square integrable, with norm:

$$\begin{array}{c} {\left\|I\right\|}_{H^{k}}^{2}=\sum_{h_{1},h_{2},h_{3}:\left|\sum_{i=1}^{3}h_{i}\right|\leq k}\int_{R^{3}}{\left|\partial_{h}I\left(x,y,z\right)\right|}^{2}dxdydz\;. \end{array}$$ (7)

This is a quadratic form for a Gaussian random field prior on the dense histology stack with zero mean and covariance dependent on the squared norm ${\left\|I\right\|}_{H^{k}}^{2}$. For the purpose of stacking, the z-axis sections are sparse 20-40 μm; the differential operators ∂_*h*_ are implemented via the difference operator along the sectioning z-axis (see Eq (8)). The Gaussian field has covariance determined by the difference operators; see [31] for example. We define the mixed differential-difference operator *D*_*h*_ as the centered difference for the z-partial derivatives,

$$\begin{array}{c} D_{h}f\left(x,y,z\right)=\partial_{h_{1},h_{2}}(\frac{f(x,y,z+\Delta /2)-f(x,y,z-\Delta /2)}{\Delta })\;. \end{array}$$ (8)

The gradient is forced to 0 at the boundaries of the image.

### MAP, penalized-likelihood reconstruction

Model the random sectioning with section-independent jitter as a product density $\pi \left(R\right)=\prod_{i}\pi \left(\theta_{i},t_{i}^{x},t_{i}^{y}\right)$, the priors centered at identity, with the priors on *θ* circular Gaussian with standard-deviation *σ*_*θ*_ and translation with means $\mu_{c}^{x},\mu_{c}^{y}$ at the center of the sections with $\sigma_{c}^{x}=\sigma_{c}^{y}$:

$$\begin{array}{c} \pi \left(\theta ,t^{x},t^{y}\right)=\frac{1}{\sqrt{2\pi }\sigma_{\theta }}e^{-\frac{\theta^{2}}{2\sigma_{\theta }^{2}}}\frac{1}{\sqrt{2\pi }\sigma_{c}^{x}}e^{-\frac{{\left(t_{x}-\mu_{c}^{x}\right)}^{2}}{2\sigma_{c}^{2}}}\frac{1}{\sqrt{2\pi }\sigma_{c}^{y}}e^{-\frac{{\left(t_{y}-\mu_{c}^{y}\right)}^{2}}{2\sigma_{c}^{2}}}\;. \end{array}$$ (9)

We choose our standard-deviations so that they are small relative to the center of the image, and a small rotation, roughly 5 percent of the total range of each. Generating MAP estimates of the rigid motions generates the MAP estimator of the histology restacking problem denoted as

$$\begin{array}{c} I^{R}\left(x,y,z_{i}\right)=J_{i}\circ R_{i}\left(x,y\right),\left(x,y\right)\in R^{2},\;\;i=1,\ldots ,n\;. \end{array}$$

Since the diffeomorphisms are infinite dimensional, the maximization of the log-likelihood function with respect to a function with the deformation penalty is termed the “penalized-likelihood estimator”. Conditioned on the known atlas, the augmented random variables to be estimated are $\left(R_{1},\ldots ,R_{n},\varphi \right)\in \left(R^{3n}\times Diff\right)$.

**Problem 1 (MAP, Penalized-Likelihood Estimator)**.

*Given histology stack* $J_{i}(x,y),(x,y)\in {\mathbb{R}}^{2},\;i=1,\ldots$ *and reconstructed stack I*^*R*^(⋅, *z*_*i*_) = *J*_*i*_ ∘ *R*_*i*_(⋅), *i* = 1, …, *n modelled as conditionally Gaussian random fields conditioned on jitter and smooth dormation of the template. The joint MAP, Penalized-Likelihood estimators arg max*_*R*,*v*_ log *π*(*R*, *v*|*J*) *given by*

$$\begin{array}{ll} {\text{argmax}}_{R,v} & -\frac{1}{2}\overset{1}{\underset{0}{\int }}\|v_{t}{\|}_{V}^{2}dt-\frac{1}{2}\underset{i}{\sum }\|D_{h}I^{R}\left(\cdot ,z_{i}\right){\|}_{2}^{2}\\ & +\underset{i}{\sum }\left(\text{log}\;\pi \left(R_{i}\right)-\alpha_{i}\|I^{R}\left(\cdot ,z_{i}\right)-I_{0}\circ \varphi^{v,-1}\left(\cdot ,z_{i}\right){\|}_{2}^{2}\right). \end{array}$$ (10)

*The MAP*, *Penalized-Likelihood estimators satisfy*

$$\{\begin{array}{l} R^{*}={\text{argmax}}_{R_{i},i=1,\ldots }\underset{i}{\sum }(\text{log}\;\pi \left(R_{i}\right)-\frac{1}{2}\|D_{h}I^{R}\left(\cdot ,z_{i}\right){\|}_{2}^{2}-\alpha_{i}\|I^{R}\left(\cdot ,z_{i}\right)-I_{0}\circ \varphi^{v^{*},-1}\left(\cdot ,z_{i}\right){\|}_{2}^{2}),\\ v^{*}={\text{argmax}}_{v}-\frac{1}{2}\overset{1}{\underset{0}{\int }}\|v_{t}{\|}_{V}^{2}dt-\underset{i}{\sum }\alpha_{i}\|I^{R^{*}}\left(\cdot ,z_{i}\right)-I_{0}\circ \varphi^{v,-1}\left(\cdot ,z_{i}\right){\|}_{2}^{2} \end{array}$$

*with* ${\left\|\cdot \right\|}_{2}^{2}$ *denoting the norm per z-axis section*:

$$\begin{array}{c} \|f\left(\cdot ,z_{i}\right){\|}_{2}^{2}=\int_{R^{2}}f{\left(x,y,z_{i}\right)}^{2}dxdy\;. \end{array}$$ (11)

We call this the **atlas-informed** model. The first two prior terms of (10) control the smoothness of template deformation and the realigned target image stack, with the third keeping the rigid motions close to the identity. The last term is the “log-likelihood” conditioned on the other variables.

The optimization for the R* rigid-motions is not decoupled across sections because of the smooth diffeomorphism of the LDDMM update and the Sobolev metric represented through the difference operator across the *z*− sections. Clearly, the smooth diffeomorphism is able to interpolate through the measured target sectioning data when the restacking solution gives a relatively smooth target, as diffeomorphisms are spatially smooth with at least one derivative. The optimization of the vector field v* corresponds to the LDDMM solution of Beg [25].

The principal algorithm used for solving this joint MAP-penalized likelihood problem alternates between fixing the rigid motions and solving LDDMM and fixing the diffeomorphism and solving for the rigid motions. This is described below in the following section.

When there is no atlas available this is equivalent to setting *α*_*i*_ small and becomes a MAP rigid motion restacking of the sections:

$$\begin{array}{c} {\text{argmax}}_{R_{i},i=1,\ldots }\sum_{i}(\log\pi \left(R_{i}\right)-\frac{1}{2}{\left\|D_{h}I^{R}\left(\cdot ,z_{i}\right)\right\|}_{2}^{2})\;. \end{array}$$

We term this the **atlas-free** model. The gradient of the rigid motions with respect to the components of translations *t*^*x*^, *t*^*y*^ and rotation *θ* is defined in S3 Text. The registration is not independent across sections due to coupling through the Sobolev metric.

### Iterative algorithm for joint penalized likelihood and MAP estimator

Here we describe the details of the algorithm used for solving for the MAP/penalized–likelihood problem described above. The algorithm alternately fixes the set of rigid motions while updating LDDMM and fixes the diffeomorphism while updating the rigid motions.

**Algorithm 1**.

*0*. *Initialize φ*^*new*^, *R*^*new*^ ← *φ*^*init*^, *R*^*init*^, *I*^*old*^ ← *J* ∘ *R*^*init*^:

*1*. *Update* $\varphi^{old}\leftarrow \varphi^{new},R_{i}^{old}\leftarrow R_{i}^{new}$, *I*^*old*^(⋅, *z*_*i*_) ← *I*^*new*^(⋅, *z*_*i*_), *i* = 1,….

*2*. *Update LDDMM for diffeomorphic transformation of atlas coordinates*:

$$\begin{array}{ccc} v^{new} & = & {\text{argmax}}_{v}\;-\frac{1}{2}\int_{0}^{1}\|v_{t}{\|}_{V}^{2}dt-\sum_{i}\alpha_{i}{\left\|I^{R-old}\left(\cdot ,z_{i}\right)-I_{0}\circ \varphi_{1}^{v-1}\left(\cdot ,z_{i}\right)\right\|}^{2}\;,\;\;\;\;\;\;\;\\ \varphi^{new} & = & \int_{0}^{1}v_{t}^{new}\circ \varphi_{t}^{new}dt+\text{id}\;\;. \end{array}$$ (12)

*3*. *Deform atlas I*_0_ ∘ *φ*^*new*−1^ *and generate new histology image stack*:

$$\begin{array}{ccc} & R^{new} & =\arg\max_{R_{i},i=1,\ldots }\sum_{i}(\log\pi (R_{i})-\frac{1}{2}\|D_{h}I^{R}\left(\cdot ,z_{i}\right){\|}_{2}^{2}-\alpha_{i}{\left\|I^{R}\left(\cdot ,z_{i}\right)-I_{0}\circ \varphi^{new-1}\left(\cdot ,z_{i}\right)\right\|}_{2}^{2})\;;\\ & I^{R-new}\left(\cdot ,z_{i}\right) & =J_{i}\circ R_{i}^{new}\left(\cdot \right)\;,i=1\ldots \end{array}$$ (13)

*4*. *Return to Step 1 until convergence criterion met*.

The form of the gradients for the rigid motions is given in S4 Text. The LDDMM update solutions are given by Beg [25].

### Software implementation

The algorithm described above is applied to Nissl histological stacks using the Allen Institute’s mouse brain atlas as a template. The Allen Mouse Brain Atlas is a micron-scale atlas that includes annotated Nissl-stained images at 10, 25, 50, and 100 μm voxel resolution, with 738 labeled compartments in the annotation.

Atlas mapping is computed on the Nissl-stained histological image stack showing the clear definition of anatomical boundaries. The associated fluorescent tracer images are transformed to the Nissl stack so that the atlas subvolume labels can be cast onto the new modality. The fluorescent and Nissl images are registered within animals by applying rigid registration based on a mutual information cost function.

**Fig 2:**
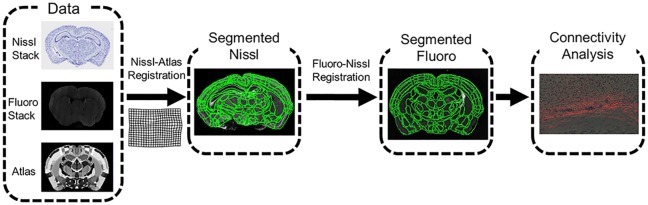
Histology registration pipeline workflow. The pipeline begins with an atlas volume and target sections, proceeds through nissl-to-atlas mapping following by fluoro-to-nissl mapping, and ends with connectivity analysis.

A software pipeline which performs start-to-finish registration operations was implemented on a high performance computing cluster for atlas-mapping and histology restacking on the Mouse Brain Architecture data. To date, the pipeline has been successfully run on over 1000 MBAP brains. The general pipeline workflow is illustrated in Fig 2. In our application, we apply a two channel LDDMM [32] algorithm for the optimization with respect to *φ*, where the first channel is the Nissl-stained grayscale image, and the second channel is a mask of the brain tissue with ventricles and background set to a pixel value of zero. The brain mask for each brain stack is automatically generated by thresholding at an estimated background intensity value and applying morphological opening and closing for denoising. The threshold value is estimated by a RANSAC-like procedure over the image histogram, assuming a normal distribution of intensity values in the image foreground. A first-order Sobolev-norm (see below) is used for the smoothness constraint regularization of the histology stack. In order to accommodate for sections damaged by the histology process or structures excluded from imaging, the objective functions in all parts of the algorithm are optimized with respect to only the image data that exists. Essentially, this is a masking procedure on the cost function that allows matching between a whole atlas brain and some target which is a partial, or subset of a whole brain.

After registration of the structural Nissl image, the fluorescence volume is registered to its corresponding Nissl volume. The registration is restricted to rigid motions on each individual section. The optimization bears a similar form to Eq (13) with the squared error matching term replaced with mutual information in order to account for the different modalities of the template and target histology stack. Once fluoro-to-Nissl registration is complete, the Nissl segmentation can be applied to the fluorescence image.

The LDDMM algorithm that maps the atlas image to an aligned stack of sections is implemented in C++. Images and other data are stored as basic arrays, and there are no dependencies other than for FFTs (we use FFTW or Intel MKL depending on availability). The remainder of code is written in Matlab (Natick, MA).

The run-time/complexity for the volume LDDMM algorithm has complexity order *n*_*T*_ *N*_*vox*_*log*(*N*_*vox*_), where *n*_*T*_ is the number of steps for integrating the time varying velocity field, and *N*_*vox*_ is the total number of voxels. The slice based portion of the code is order *N*_*vox*_. While the FFTs are order *NlogN*, in practice most computation time is spent during linear interpolation (order *N*). The end-to-end running time from initial stack alignment to completed atlas registration is approximately 6-8 hours using 8 cores on an Intel Xeon E5-2665 processor for target and template image volumes of approximately 200 × 300 × 300 voxels. Jobs are performed in parallel on a high performance cluster at CSHL. The fluoro-to-nissl cross registration running time is approximately 1 hour on the same environment and volume size.

The following hyper-parameters are required by our model, with sample values provided for the MBAP dataset:

1. the weights between the matching term (1.0), the regularizing prior (0.001), and the Sobolev norm (1.0) on the rigid objective function
2. the variances of the priors on rotation ($\frac{\pi }{9}$) and translation (7.0) in each stacking plane
3. the weight between the matching term (0.4) and the regularizing term in LDDMM (1.0)
4. the LDDMM kernel size (a cascade of 0.05, 0.02, and 0.01)
5. the initial gradient descent step size (0.000025 for rigid parameters and 5e-13 for LDDMM parameters)

The hyper-parameters were selected by grid search on a predefined range of parameter values, testing the rigid stack alignment and LDDMM parameters separately.

## Results and discussion

### Validation on simulated reconstructions

#### Binary phantom with curvature distortion

**Fig 3:**
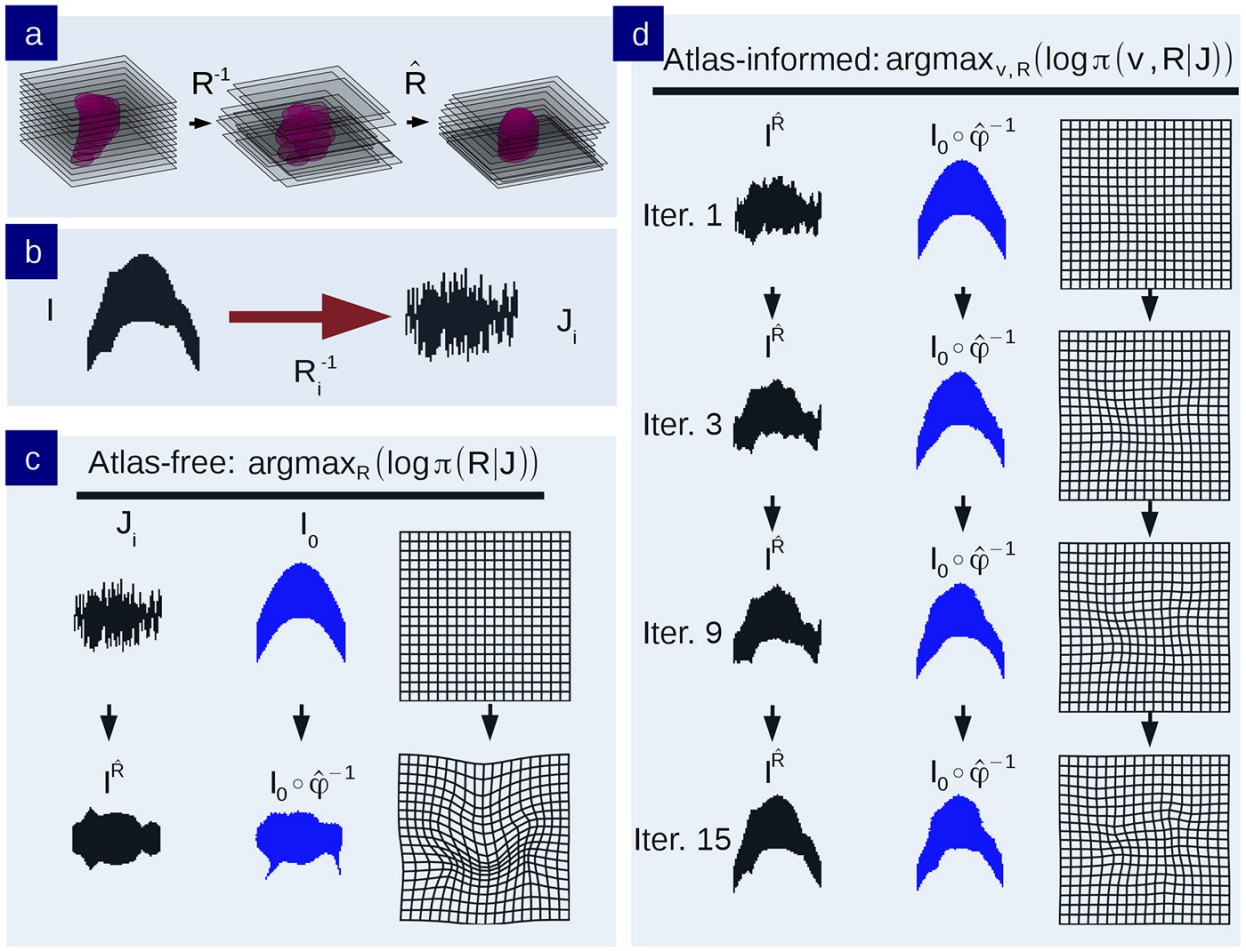
Comparison of atlas-free and atlas-informed models in simulated binary phantom. a) An illustration of the classic curvature reconstruction problem. b) The unobserved 3D-phantom is randomly sectioned and observed as *J*_*i*_, *i* = 1, …, *n*. c) Reconstruction of the histological stack using the atlas-free method. The top row shows the histological stack and atlas. The bottom row shows the reconstructed histological stack $I^{\hat{R}}$ alongside the deformed phantom atlas *I* = *I*_0_ ∘ *φ*^−1^ which has been mapped to histological sections, and the diffeomorphic change of coordinates ${\hat{\varphi }}^{-1}$. d) Reconstruction of phantom using the atlas-informed model. Each row depicts iterations of the reconstructed histological stack $I^{\hat{R}}$ alongside the deformed atlas $I=I_{0}\circ {\hat{\varphi }}^{-1}$ and deformed coordinates. The bottom row is the convergence point of the algorithm.

The model was applied to binary image phantoms in order to examine the “curvature” problem in which a 3D curved object cannot be accurately reconstructed after being sectioned. This is illustrated in Fig 3. We produced sections through the 3D phantom, applying the atlas-free and the atlas-informed models. The results from the atlas-free algorithm in which the sections are aligned based on the Sobolev smoothness followed by mapping of the atlas via LDDMM are summarized in Fig 3c. The atlas-free section alignment reconstructs the target stack, demonstrating a cylindrical reconstruction rather than the curved template shape, followed by LDDMM alignment *I*_0_ ∘ *φ*^−1^. This illustrates the curvature issue. The atlas coordinate grid is transformed significantly (bottom right of Fig 3c) in order to match the target. Despite this significant deformation, there is some residual error in the atlas-to-target mapping with the remaining tendrils where the ends of the phantom did not shrink inwards. Here, the energy required to push the ends of the atlas inwards was greater than the potential image matching improvement.

Shown in Fig 3d is the atlas-informed solution. The bottom row shows that simultaneously solving for reconstruction and registration parameters allows for more consistent stack reconstruction of the target resulting from the influence of the smooth deformation of the template onto the target in the joint solution.

**Fig 4:**
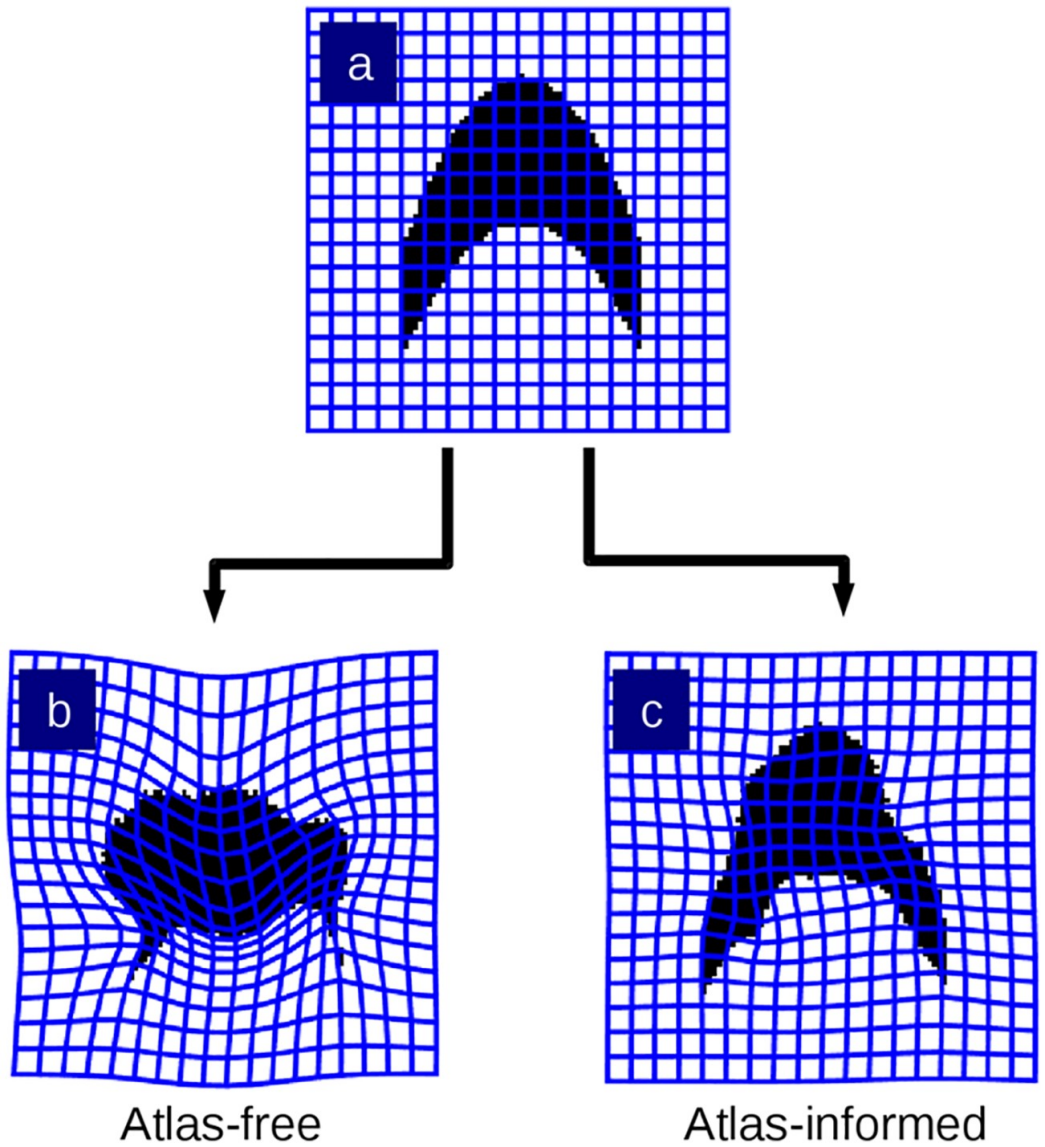
Comparison of resulting diffeomorphic transformation of atlas phantoms. The warped coordinate grids illustrate the difference in the mapping deformation from the atlas-free methods from (A) to histology stack target (B) versus the atlas-informed algorithm which produces (C).

These results are depicted by the motions of the atlas coordinate grids when deforming onto the targets in Fig 4. Tandem optimization of section alignment parameters and diffeomorphisms produces a nonlinear mapping with lower metric cost (Fig 4c is less warped than Fig 4b).

#### Simulated jitter on the Allen atlas

**Fig 5:**
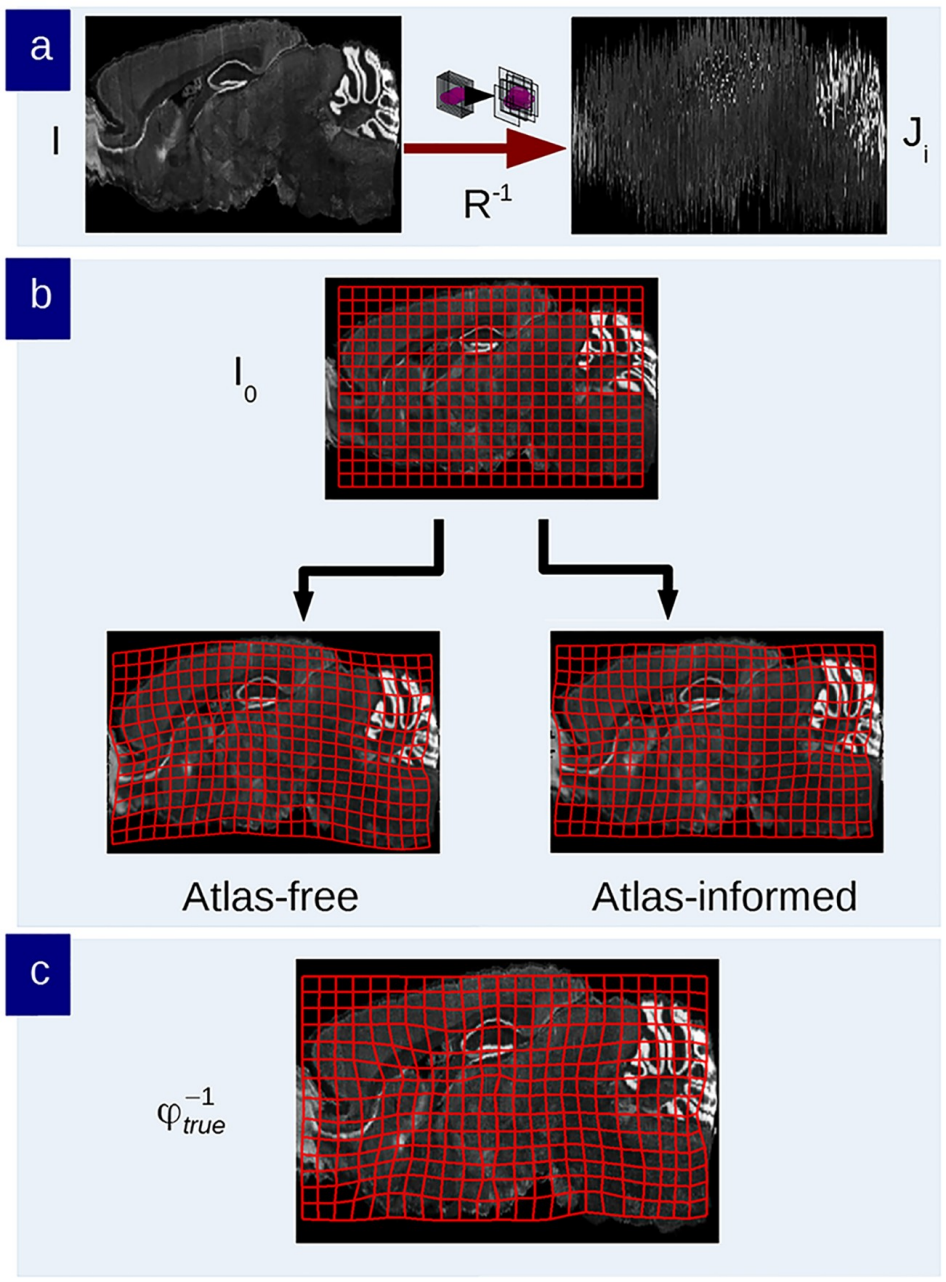
Atlas phantom simulation to validate recovery of sectioning parameters and diffeomorphic shape difference. a) The ground truth target *I* is sectioned to generate the observed target *J*_*i*_. b) Transformed grids illustrating the brain phantom atlas (top) shown mapped onto the histological stack using the atlas-free algorithm (bottom left) and the atlas-informed algorithm (bottom right). c) The ground truth diffeomorphism to be recovered.

A similar experiment was performed using the Allen mouse brain atlas as the 3D phantom. A target histology stack was generated by sectioning the Allen atlas in simulation and applying random rigid transforms to its coronal sections. The atlas images were sampled at 40 μm isotropic voxels. This is depicted in Fig 5a. A simulated atlas was generated by applying a given random diffeomorphism to the Allen atlas. This random diffeomorphism is depicted in Fig 5c. The histology stacks were then reconstructed and diffeomorphic transformations generated between the atlas and target stacks using both models, intending to recover both the unknown rigid transforms from Fig 5a and the unknown diffeomorphism from Fig 5c. Fig 5b shows the atlas-free method method (bottom left) compared to the atlas-informed method (bottom right). The atlas-informed method nearly reproduces the original coordinates whereas the atlas-free method drifts away from the original coordinates. Note that although the diffeomorphisms are not identical, this does not necessarily indicate segmentation error as small differences in stack alignment can be compensated for by nonlinear registration during atlas-mapping.

#### Simulated bias and variance statistics

**Fig 6:**
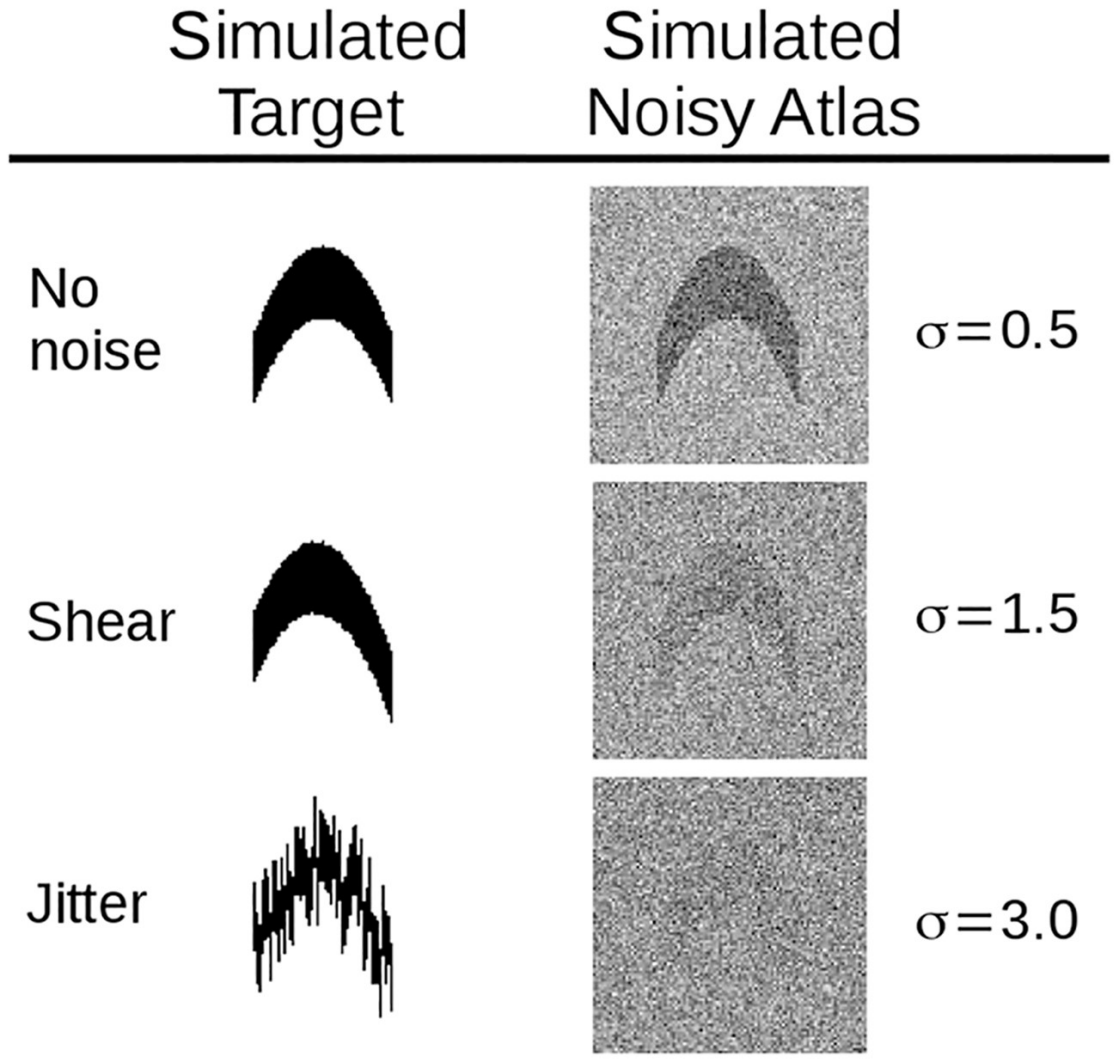
Simulated noise on a binary image phantom. Left column shows phantom for identity, shearing, and jitter of sections (successive rows); right column shows Gaussian white noise added to the atlas at various standard deviations.

**Fig 7:**
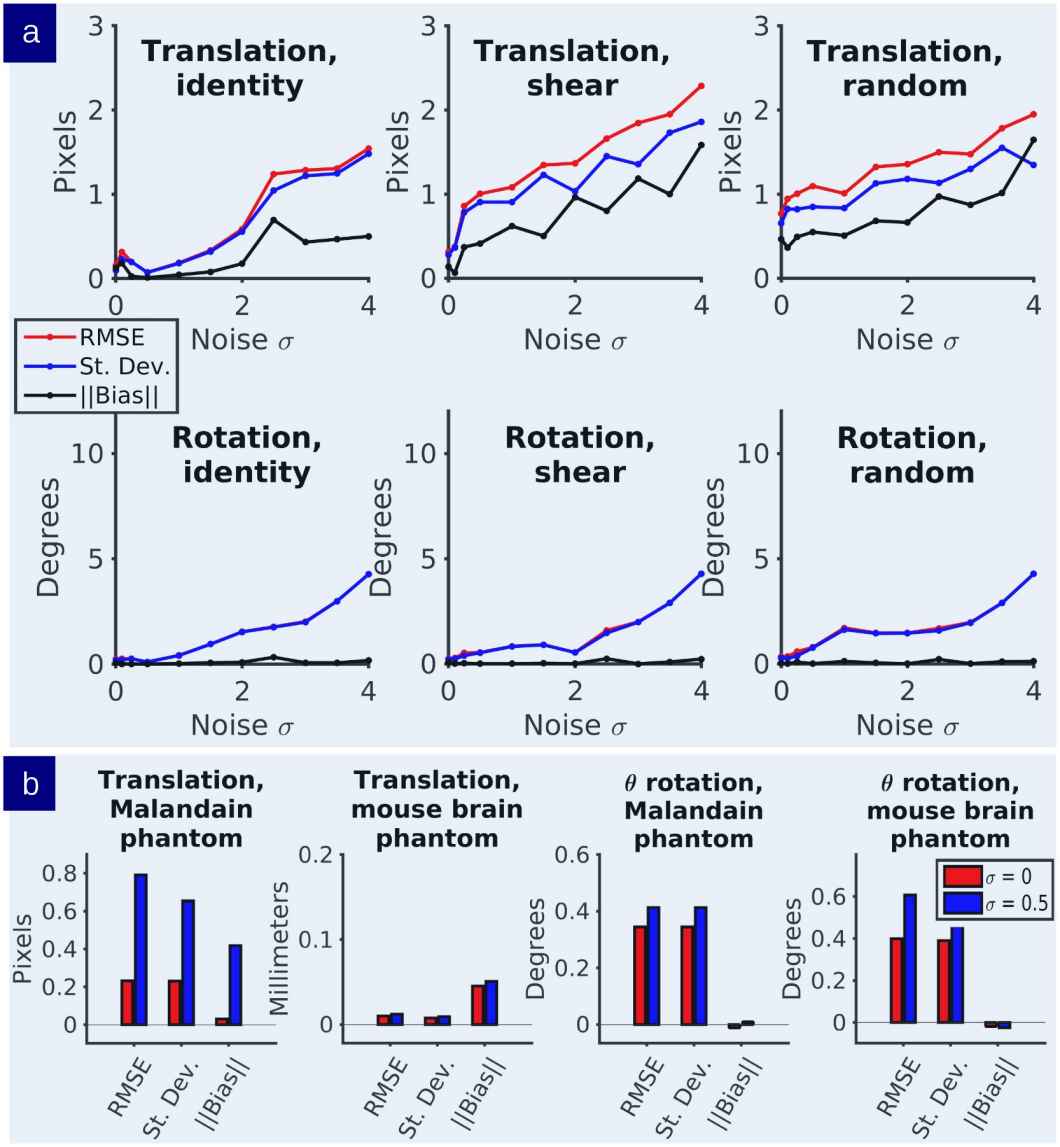
Evaluation of estimator MSE, variance, and bias. a) Statistics on the translation-rotation estimators for noise levels varying initial conditions. b) Statistics on the rigid motion estimators where the section jitter was added in a random fashion.

Figs 6 and 7 show results quantifying the bias and viarance of the joint estimation of the diffeomorphism transformation and the rigid motion jitter in simulation. Eq (2) was simulated over a range of Gaussian white noise selections while simultaneously varying the jitter rigid motions of the sections along with multiple deformations of shearing applied to the template *I*_0_. Shearing produced images where each section was successively offset by 0.25 pixels in both x and y directions, cumulatively producing the “shear” effect illustrated in Fig 6. Fig 7a keeps the stack jitter fixed and varies the noise levels; Fig 7b varies the stack jitter. The random rigid motion jitter was normally distributed $\left(t_{x},t_{y}\right)∼N\left(\mu =0,\sigma^{2}=36\right),\theta ∼N\left(\mu =0,\sigma^{2}=100\right)$ in pixel units. The RMSE, bias, and standard deviation of the estimated parameters were computed in each experiment and plotted as a function of error units versus noise level. 500 simulations per experiment were performed.

In each experiment, estimator accuracy is preserved up to high noise levels. At typical noise levels (*σ* ≤ 0.5), we observe subpixel RMSE and small bias. Fig 7b shows that the rotation estimator is virtually unbiased whereas the translation estimator does have small subvoxel bias. It is likely that more rotational error is accounted for by section realignment than deformable mapping, whereas both play a relatively balanced role in translation correction. Small motions are ill-posed in that small rigid-motions can accommodate small atlas deformation. Fig 7c (top row) shows the case where there is jitter in the target stack. Estimator statistics are computed in each of these cases showing similar subpixel errors.

A similar analysis was performed for the Allen atlas brain phantom simulations. The reconstruction RMSE observed in the brain phantom simulation (bottom row of Fig 7c) is lower than that observed in the simple curved phantom in pixels. It is likely that this is due to the presence of more contour lines in grayscale images versus binary images. These additional features allow for more accurate distinction of matching error than simpler images with small numbers of distinct level lines. This is consistent with the demonstration in [27] showing that the stabilizer of the group corresponding to vector fields tangent to the level lines of the image cannot be uniquely identified or retrieved via any mapping methods that look at color or contrast of the image as the identifying feature.

### Mouse Brain Architecture Project data

A final experiment was conducted on brain data sampled from the MBAP database, using the Allen mouse brain as the atlas. We selected specific targets which were prone to poor registration results due to image intensity local minima. In particular, structures like the cerebellum tend to be difficult to register accurately due to their folded nature; one fold can easily be mistaken for the adjacent fold, and if the target and atlas are not well initialized, the deformation required to flow one fold onto another can have a high metric cost. We are also interested in inspecting lower-contrast structures like the corpus callossum, which may be poorly registered due to local minima in other nearby bright structures. We also evaluate our mapping quality in the hippocampal region, which is one of the most relevant regions for the study of neurodegenerative diseases.

**Fig 8:**
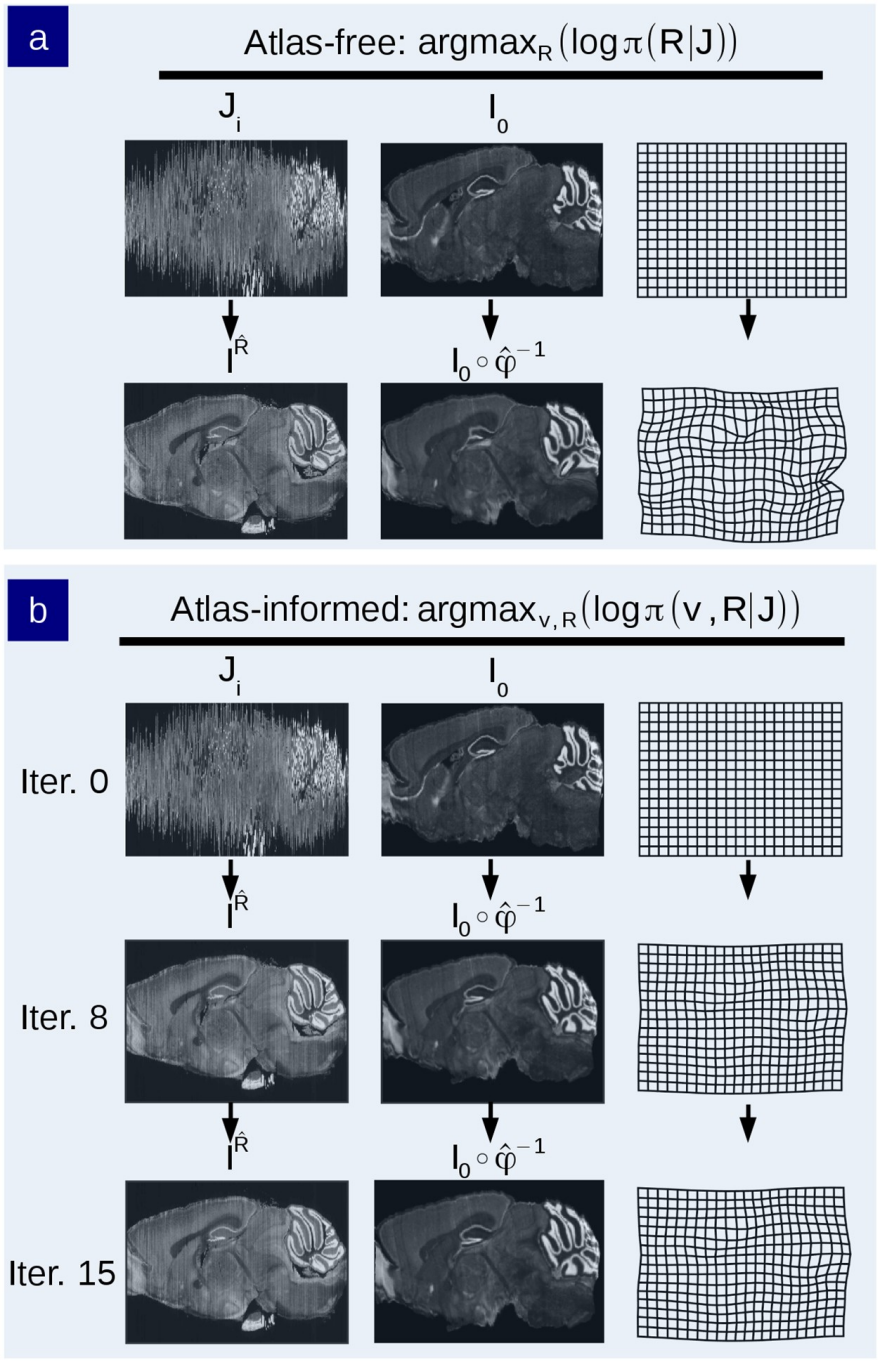
Comparison of reconstruction and mapping using atlas-free and atlas-informed models on data from the MBAP database. a) Reconstruction of an MBA Nissl-stained brain histological stack using the atlas-free method. Top row shows the histological stack and Allen mouse brain atlas. Bottom row shows the reconstructed histological stack $I^{\hat{R}}$ alongside the deformed phantom atlas *I*, and the diffeomorphic change of coordinates ${\hat{\varphi }}^{-1}$. b) Reconstruction using the atlas-informed method. Top row shows the histological stack and Allen mouse brain atlas. Middle row depicts intermediate iterations of the reconstructed stack $I^{\hat{R}}$ alongside the deformed atlas $I_{0}\circ {\hat{\varphi }}^{-1}$ and coordinate grid. Bottom row shows the convergence point of algorithm.

**Fig 9:**
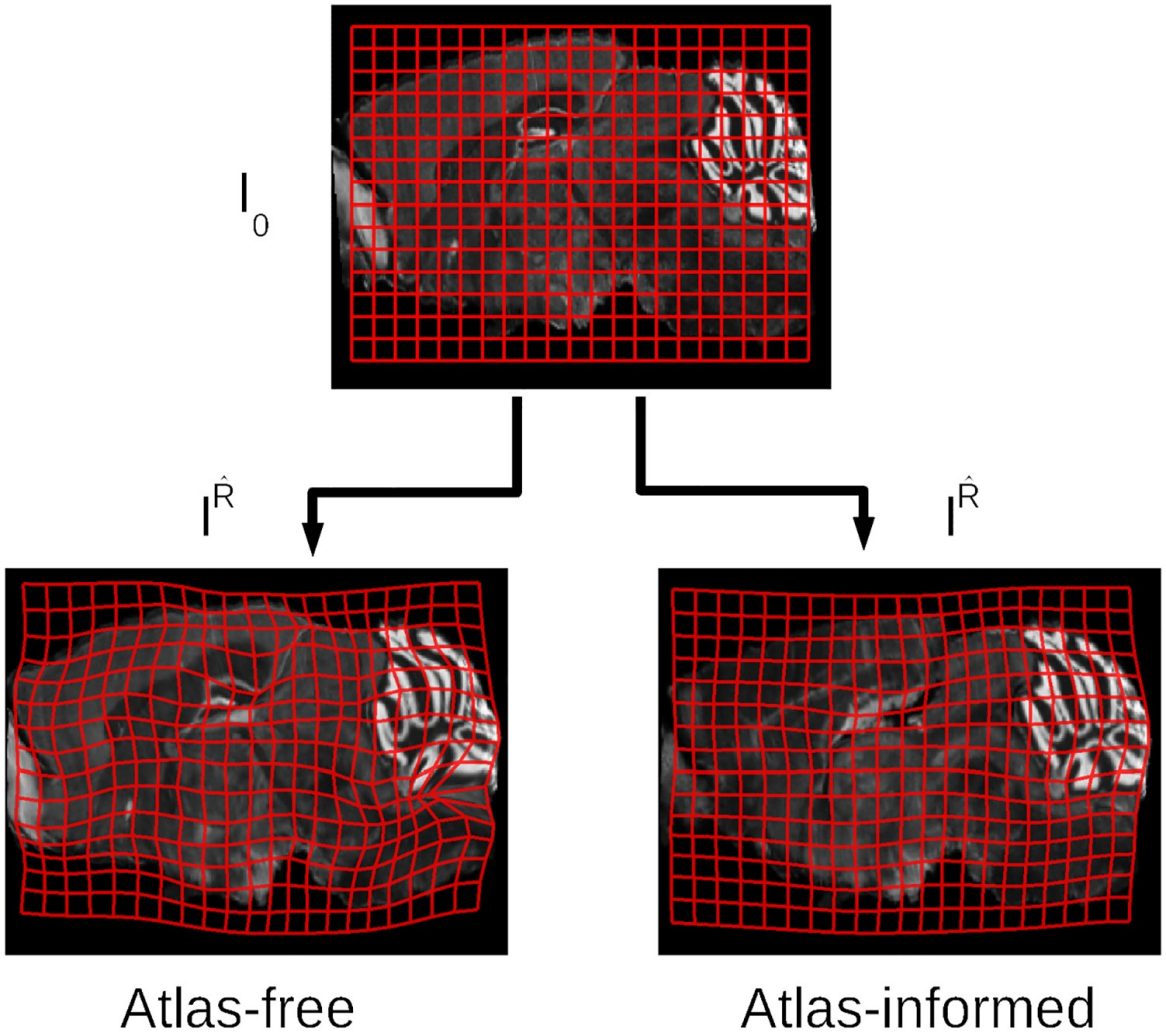
Comparison of diffeomorphic transformation recovered from atlas-free and atlas-informed models. The warped grids illustrate the difference in the mapping deformation from atlas (top) to target using the atlas-free method (bottom left) versus the atlas-informed method (bottom right), performed on real brain data from the MBA Project.

The reconstructed histological target stack in the atlas-informed model shown in Fig 8a takes on the shape of the atlas but is prone to reconstruction artifacts. The deformation grids produced by the atlas-informed mapping is much smoother and has many fewer wrinkles than the atlas-free mapping. This is seen clearly in Fig 9.

**Fig 10:**
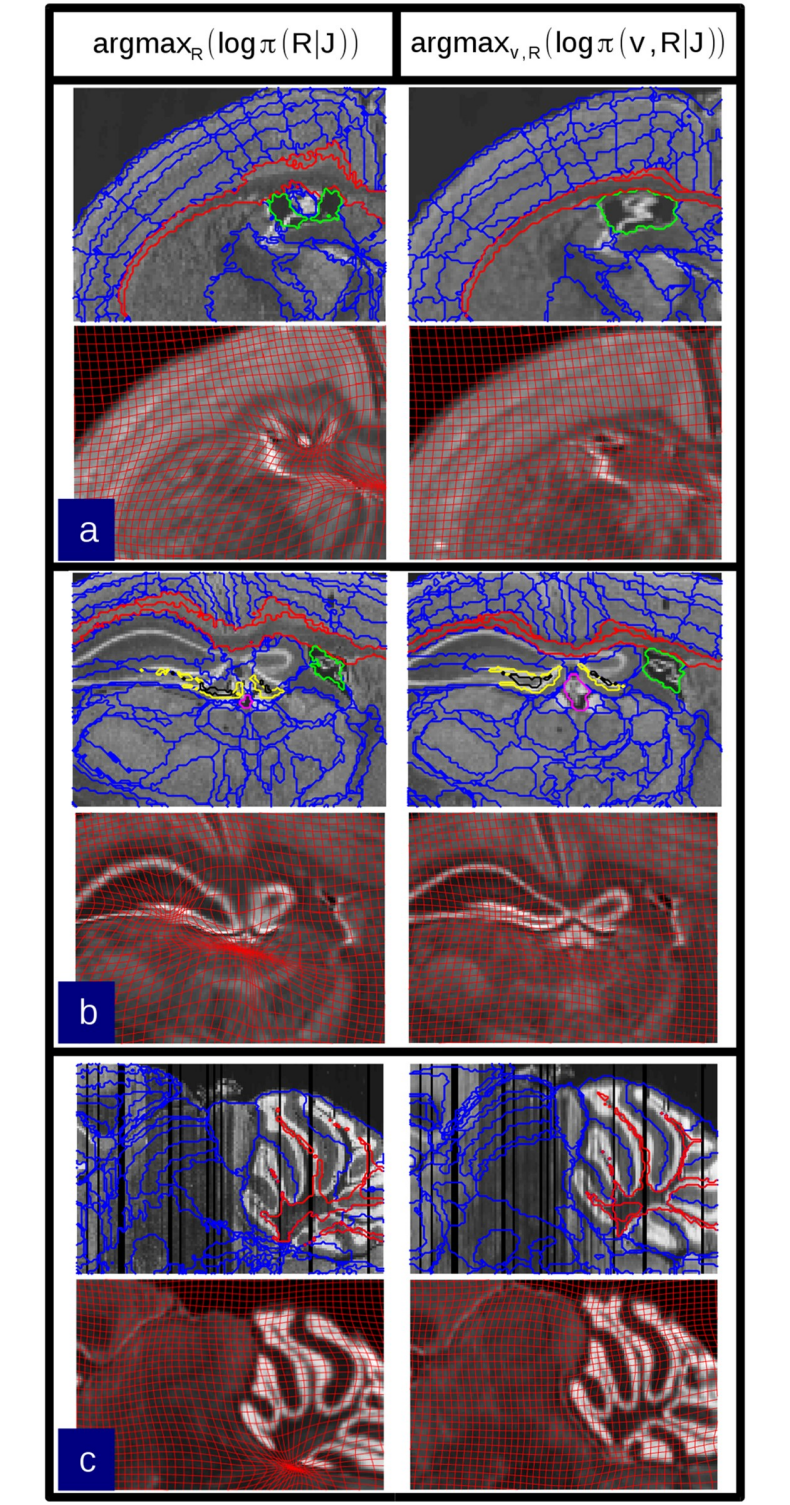
Selected regions of the brain segmented by the atlas-informed and atlas-free models carry the label map from the Allen atlas under the computed diffeomorphism. The left column shows several examples where optimization of the atlas-free solution is trapped in false minima due to folded or low-contrast structures. The right column shows correction by the atlas-informed algorithm. A) The corpus callossum and lateral ventricle. B) The dentate gyrus, corpus callossum, and lateral ventricle. C) The cerebellar white matter.

Fig 10 shows examples of improved segmentations in selected regions of the brain. The atlas-informed model generates more accurate segmentation results and produces smoother mappings as exhibited by the less wrinkled and distorted grids (bottom row b), showing more consistent results throughout the MBAP dataset.

### Conclusion

This paper examines the CA random orbit model at the mesoscale for the stacking of sectioned whole brains coupled with mapping to annotated atlases. The standard CA model has been expanded to include the O(3 × *n*) extra rigid motion dimensions representing the planar histology sections. The estimation procedure solved here simultaneously estimates the diffeomorphic change of coordinates between atlas and target histological stack, as well as the “nuisance” rigid motion parameters for each section in stack space. This requires the introduction of a smoothness constraint on the target jitter simultaneous with LDDMM, which is enforced via a Sobolev metric, encouraging the reconstructed stack to be smooth by controlling the derivative along the cutting axis.

Results are shown demonstrating that the introduction of an atlas into the estimation scheme and simultaneously accommodating for the nonlinear atlas-to-target shape difference via diffeomorphism solves several of the classic problems associated with volume reconstruction, including the recovery of the curvature of extended structures. Since the atlas gives *a priori* indication of the global shape, the tendency to remove distortions along the section axis is balanced against the desire to minimize the amount of deformation of the atlas onto the reconstruction. The algorithm is shown to mediate this tension well.

The clear limitation of this method is that we model sections that are out of order, folded upon themselves, or damaged by censoring from the mapping solution using the weighting coefficient *α*_*i*_ and removing their impact from the overall deformation. This is a global censoring, but we do not apply shearing deformations within plane and we do not include in the algorithm an automatic solution to detecting the folding problem. Although we do not currently include correction beyond rigid motion within the plane of each section, one could imagine attempting to add such shearing distortions to the model, which would remain stable as the number of new dimensions would remain low. The global censoring solution requires human quality control within the pipeline for detection of globally deformed or damaged sections.

The use of dense large deformation diffeomorphic image matching is being used extensively for magnetic resonance imaging in the brain at 1 millimeter scale for both T1 and DTI [23, 25, 32, 33] as well as for human anatomy [22] including for transferring the geometries of Cardiac fibers in dense Cardiac imaging [34, 35] and for radiation treatment planning [36]. These technologies form the basis of many implementations such as Ashburner’s important SPM [37, 38]. The aforementioned applications have not included complex prior distributions to encode distortions such as the Sobolev derivative prior introduced here that may have be required due to the distortions introduced in the imaging and stacking process.

## Supporting information

S1 Text Reproducing Kernel Hilbert Space and Green’s Kernel. (PDF)

S2 Text Geodesics solving Euler-Lagrange equations. (PDF)

S3 Text Gradients for Atlas free model. (PDF)

S4 Text Gradients for Atlas informed model. (PDF)
