# Supplementary material for: On variational solutions for whole brain serial-section histology using a Sobolev prior in the computational anatomy random orbit model
Source: PLoS Comput Biol. 2018 Dec 26;14(12):e1006610. doi: 10.1371/journal.pcbi.1006610 (PMC6324828; doi:10.1371/journal.pcbi.1006610)
Supplement: S1 Text — (PDF) [file pcbi.1006610.s001.pdf]

S1 Text – Reproducing Kernel Hilbert Space and Green's Kernel. The Green's kernel is translation invariant and takes the form

$$K(x, y, z) = k(x, y, z)Id_3 \ ,$$

with Id_3 the 3×3 identity matrix, for the Green's function continuously differentiable:

$$k(x, y, z) = 4\left(3 + 3\sqrt{x^2 + y^2 + z^2} + 3(x^2 + y^2 + z^2)\right) e^{-\sqrt{x^2+y^2+z^2}} \ .$$

This Green's function satisfies $(-\nabla^2 + 1)^4 k(x, y, z) = \delta(x, y, z)$, where $(-\nabla^2 + 1)^4$ is referred to as A. The reproducing kernel Hilbert space (RKHS) with this Green's kernel corresponds to vector fields satisfying

$$\|v\|_V^2 = \sum_{i=1}^{3} \int_{\mathbb{R}^3} ((-\nabla^2 + 1)^2 v_i(x, y, z))^2 dxdydz < \infty \ .$$
