# Supplementary material for: On variational solutions for whole brain serial-section histology using a Sobolev prior in the computational anatomy random orbit model
Source: PLoS Comput Biol. 2018 Dec 26;14(12):e1006610. doi: 10.1371/journal.pcbi.1006610 (PMC6324828; doi:10.1371/journal.pcbi.1006610)
Supplement: S2 Text — (PDF) [file pcbi.1006610.s002.pdf]

S2 Text – Geodesics solving Euler-Lagrange Equations. The explicit equations for geodesics associated to the RKHS norm $\|v\|_V$ and the geodesics satisfy the Euler-Lagrange equations [27, 30] given by the triple of equations.

$$\begin{cases} \dot{\varphi}_t = v_t \circ \varphi_t \\ \dot{p}_t = -(dv_t)^T \circ \varphi_t p_t \\ v_t = \int_{\mathbb{R}^3} K(x, \varphi_t(y)) p_t(y) dy \ , \ Av_0 = p_0 \ . \end{cases} \tag{1}$$

To prove the Hamiltonian momentum evolution, the second equation $\dot{p} = -(dv)^T \circ \varphi p$ of (1) for Av a classical function we use the inner product notation $\langle \cdot, \cdot \rangle$ to calculate the Lagrangian:

$$L(\varphi, \dot{\varphi}) = \frac{1}{2} \langle A\dot{\varphi} \circ \varphi^{-1}, \dot{\varphi} \circ \varphi^{-1} \rangle = \frac{1}{2} \int_{\mathbb{R}^3} A(\dot{\varphi} \circ \varphi^{-1}(x)) \cdot \dot{\varphi} \circ \varphi^{-1}(x) dx \ ,$$

with the variation giving the Euler-Lagrange equations:

$$\frac{d}{dt} \underbrace{\partial_{\dot{\varphi}} L(\varphi, \dot{\varphi})}_{\text{Ham. mom. p}} - \partial_{\varphi} L(\varphi, \dot{\varphi}) = 0.$$

To get the Hamiltonian momentum $p = \partial_{\dot{\varphi}} L(\varphi, \dot{\varphi})$, we take variation with respect to Lagrangian velocity $\dot{\varphi} \to \dot{\varphi}^{\varepsilon} = \dot{\varphi} + \varepsilon \delta \dot{\varphi}$ and $\varphi \to \varphi + \varepsilon \delta \varphi$ giving

$$\begin{aligned} \frac{d}{d\varepsilon} L(\varphi^{\varepsilon}, \dot{\varphi}^{\varepsilon})|_{\varepsilon=0} &= \frac{d}{d\varepsilon} \frac{1}{2} \langle A(\dot{\varphi}^{\varepsilon} \circ \varphi^{-1}), \dot{\varphi}^{\varepsilon} \circ \varphi^{-1} \rangle|_{\varepsilon=0} \\ &= \frac{d}{d\varepsilon} \frac{1}{2} \left(\langle Av, \dot{\varphi}^{\varepsilon} \circ \varphi^{-1} \rangle + \langle A(\dot{\varphi}^{\varepsilon} \circ \varphi^{-1}), v \rangle \right) |_{\varepsilon=0} \end{aligned}$$

Combining gives the Hamiltonian momentum :

$$\langle Av, \frac{d}{d\varepsilon} (\dot{\varphi} + \varepsilon \delta \dot{\varphi}) \circ \varphi^{-1} \rangle \quad = \quad \langle \underbrace{Av \circ \varphi |d\varphi|}_{\partial_{\dot{\varphi}} L \text{Ham. mom.}}, \delta \dot{\varphi} \rangle \ .$$

The variation $\varphi \to \varphi^{\varepsilon} = \varphi + \varepsilon \delta \varphi$ requires the inverse:

$$(\varphi^{-1} + \varepsilon \delta \varphi^{-1}) \circ (\varphi + \varepsilon \delta \varphi) \simeq \text{id} + \varepsilon (d\varphi^{-1})_{|\varphi} \delta \varphi + \varepsilon \delta \varphi^{-1}_{|\varphi}$$

which gives first order perturbation

$$\delta \varphi^{-1} = -(d\varphi^{-1}) \delta \varphi_{|\varphi^{-1}} = -(d\varphi)^{-1}_{\varphi^{-1}} \delta \varphi_{|\varphi^{-1}} \ . \tag{2}$$

Taking a similar variation of the Lagrangian as above but with respect to the Lagrangian velocity gives

$$\begin{aligned} \langle Av, \frac{d}{d\varepsilon} (\dot{\varphi} \circ (\varphi^{-1} - \varepsilon (d\varphi)^{-1}_{|\varphi^{-1}} \delta \varphi_{|\varphi^{-1}})) \rangle &= -\langle Av, (dv)(d\varphi)_{|\varphi^{-1}} (d\varphi)^{-1}_{|\varphi^{-1}} \delta \varphi_{|\varphi^{-1}} \rangle \\ &= -\langle \underbrace{(dv)^T_{\varphi} Av \circ \varphi |d\varphi|}_{\partial_{\varphi} L}, \delta \varphi \rangle \end{aligned} \tag{3}$$

The third equation of (1) follows from $p = Av \circ \varphi |d\varphi|$. Integrating with the Green's kernel gives the expression $v_t(\cdot) = \int K(\cdot, \varphi_t(y)) p_t(y) dy$.
