# Supplementary material for: On variational solutions for whole brain serial-section histology using a Sobolev prior in the computational anatomy random orbit model
Source: PLoS Comput Biol. 2018 Dec 26;14(12):e1006610. doi: 10.1371/journal.pcbi.1006610 (PMC6324828; doi:10.1371/journal.pcbi.1006610)
Supplement: S3 Text — (PDF) [file pcbi.1006610.s003.pdf]

**S3 Text – Gradients for Atlas Free Model** We can write the gradient with respect to the components of $R$ (translation vector $t$ and rotation matrix $r$ parametrized by rotation angle $\theta$ and section number $z$), where $\nabla_X$ is the 2D in-plane gradient, $\sigma_{JJ}$ is the weighting factor on the image smoothness prior. Rotations and translations are penalized by a regularization prior centered at identity ($\frac{\theta}{\sigma_\theta^2}$ and $\frac{t(z_i)}{\sigma_t^2}$, respectively), where $\sigma_\theta$ and $\sigma_t$ are weighting factors on the rotation and translation priors arising as standard-deviations of the Gaussian priors written out in Eqn. (9).

$$\nabla_r E = -\alpha(z_i)\frac{d^2}{dz^2}\left(J(r(\theta, z_i)x + t(z))\right)\nabla_X J(r(\theta, z_i)x + t(z_i))\delta r(\theta, z_i)x + \frac{\theta}{\sigma_\theta^2} \quad (1)$$

$$\nabla_t E = -\alpha(z_i)\frac{d^2}{dz^2}\left(J(r(\theta, z)x + t(z_i))\right)r(\theta, z), \nabla_X J(r(\theta, z_i)x + t(z_i)) + \frac{t(z_i)}{\sigma_t^2} \quad (2)$$

Here the weight $\alpha_i$ plays the role of controlling the step size in the gradient algorithm rather than controlling the weight relative to the prior of the likelihood function as it does in the atlas-informed case. For image planes that are noisy, the step-size is small, approximately zero.
