# Supplementary material for: On variational solutions for whole brain serial-section histology using a Sobolev prior in the computational anatomy random orbit model
Source: PLoS Comput Biol. 2018 Dec 26;14(12):e1006610. doi: 10.1371/journal.pcbi.1006610 (PMC6324828; doi:10.1371/journal.pcbi.1006610)
Supplement: S4 Text — (PDF) [file pcbi.1006610.s004.pdf]

S4 Text – Gradients for Atlas Informed Model The minimization of the energy E_v of (12) in terms of the vector field is the LDDMM gradient of Beg [25]:

$$\nabla_v E_v(x,y) = \sum_i \int_{\mathbb{R}^2} K(x-x', y-y', z-z_i)|D\varphi_{t,1}| \left(I\circ\varphi_{t1} - I_0\circ\varphi_t^{-1}\right) \nabla(I_0\circ\varphi_t^{-1})(x',y',z_i)\big)\, dx'dy' \ . \quad (1)$$

Variation of the Image Matching Term: The variation of $\int(I-I_0\circ\varphi^{-1})^2dx$ via perturbation $\varphi\to\varphi^\varepsilon=\varphi+\varepsilon\delta\varphi$ requires the inverse perturbation $\delta\varphi^{-1}=-(d\varphi)^{-1}_{\varphi^{-1}}\delta\varphi|_{\varphi^{-1}}$, derived in (2) above. Then we have

$$\begin{aligned}\frac{d}{d\varepsilon}\int_{\mathbb{R}^3}(I-I_0\circ\varphi^{\varepsilon-1})^2dx|_{\varepsilon=0} &= 2\int_X (I-I_0\circ\varphi^{-1})\nabla(I_0)|\varphi^{-1}\cdot(d\varphi)^{-1}_{|\varphi^{-1}}\delta\varphi_{|\varphi^{-1}})dx \\ &= 2\int_X (I\circ\varphi-I_0)(d\varphi)^{-1T}\nabla I_0|d\varphi|\cdot\delta\varphi dx \ .\end{aligned}$$

Rigid motion variations: Rigid motion minimization is standard for rigid registration in 2D and 3D images. Denoting $\|f_{\theta,t,z_i}\|^2 = \|J^R(\cdot,z_i)-I_0\circ\varphi^{v^*-1}(\cdot,z_i)\|_2^2$ to represent each rigid registration norm-square minimization within each histological plane, then

$$\nabla_\theta\|f_{\theta,t,z_i}\|^2 = \int_{\mathbb{R}^2} 2f_{\theta,t,z_i}(\cdot)\frac{\partial_\theta f_{\theta,t,z_i}}{\partial\theta}dxdy \ ;$$

$$\nabla_t\|f_{\theta,t,z_i}\|^2 = \int_{\mathbb{R}^2} 2f_{\theta,t,z_i}(\cdot)\nabla_t f dxdy \ .$$

$$\nabla_{R,t}\ell(v,R;J) = \Big\langle \frac{1}{\sigma_{JI}^2}(I_{\varphi^{-1}}(x)-J(r(\theta,z)x+t(z))) - \frac{1}{\sigma_{JJ}^2}\frac{d^2}{dz^2}\left(J(r(\theta,z)x+t(z))\right)r(\theta,z), \nabla_X J(r(\theta,z)x+t(z))\Big\rangle + \frac{t(z)}{\sigma^2_{reg_t}} \quad (2)$$

$$\nabla_{R,r}\ell(v,R;J) = \Big\langle \frac{1}{\sigma_{JI}^2}(I_{\varphi^{-1}}(x)-J(r(\theta,z)x+t(z))) - \frac{1}{\sigma_{JJ}^2}\frac{d^2}{dz^2}\left(J(r(\theta,z)x+t(z))\right), \nabla_X J(r(\theta,z)x+t(z))R\begin{bmatrix}0 & 1\\ -1 & 0\end{bmatrix}x\Big\rangle + \frac{\theta}{\sigma^2_{reg_r}} \quad (3)$$

where σ_{JI} is a weighting factor on the matching term between atlas and target.
